# Carbon nanodot–based electrogenerated chemiluminescence biosensor for miRNA-21 detection

**DOI:** 10.1007/s00604-021-05038-y

**Published:** 2021-10-30

**Authors:** Laura Gutiérrez-Gálvez, Tania García-Mendiola, Cristina Gutiérrez-Sánchez, Tamara Guerrero-Esteban, Cristina García-Diego, Irene Buendía, M. Laura García-Bermejo, Félix Pariente, Encarnación Lorenzo

**Affiliations:** 1 Department of Analytical Chemistry and Instrumental Analysis, Universidad Autónoma de Madrid, Ciudad Universitaria de Cantoblanco, 28049 Madrid, Spain; 2 Institute for Advanced Research in Chemical Sciences (IAdChem), Universidad Autónoma de Madrid, Ciudad Universitaria de Cantoblanco, 28049 Madrid, Spain; 3 IMDEA Nanociencia, Ciudad Universitaria de Cantoblanco, 28049 Madrid, Spain; 4 Instituto de Catálisis y Petroleoquímica, Consejo Superior de Investigaciones Científicas, C/Marie Curie 2, 28049 Madrid, Spain; 5 Biomarkers and Therapeutic Targets Group and Core Facility, Instituto Ramón y Cajal de Investigación Sanitaria (IRYCIS), Spanish Renal Research Network (REDinREN), Madrid, Spain

**Keywords:** Green chemistry synthesis, Carbon nanomaterials, RNA detection, ECL biosensor

## Abstract

A simple carbon nanodot–based electrogenerated chemiluminescence biosensor is described for sensitive and selective detection of microRNA-21 (miRNA-21), a biomarker of several pathologies including cardiovascular diseases (CVDs). The photoluminescent carbon nanodots (CNDs) were obtained using a new synthesis method, simply by treating tiger nut milk in a microwave reactor. The synthesis is environmentally friendly, simple, and efficient. The optical properties and morphological characteristics of the CNDs were exhaustively investigated, confirming that they have oxygen and nitrogen functional groups on their surfaces and exhibit excitation-dependent fluorescence emission, as well as photostability. They act as co-reactant agents in the anodic electrochemiluminescence (ECL) of [Ru(bpy)_3_]^2+^, producing different signals for the probe (single-stranded DNA) and the hybridized target (double-stranded DNA). These results paved the way for the development of a sensitive ECL biosensor for the detection of miRNA-21. This was developed by immobilization of a thiolated oligonucleotide, fully complementary to the miRNA-21 sequence, on the disposable gold electrode. The target miRNA-21 was hybridized with the probe on the electrode surface, and the hybridization was detected by the enhancement of the [Ru(bpy)_3_]^2+^/DNA ECL signal using CNDs. The biosensor shows a linear response to miRNA-21 concentration up to 100.0 pM with a detection limit of 0.721 fM. The method does not require complex labeling steps, and has a rapid response. It was successfully used to detect miRNA-21 directly in serum samples from heart failure patients without previous RNA extraction neither amplification process.

**Graphical abstract:**

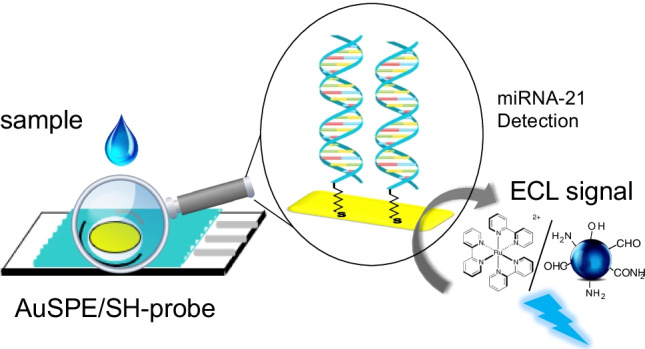

**Supplementary Information:**

The online version contains supplementary material available at 10.1007/s00604-021-05038-y.

## Introduction

Carbon nanomaterials are currently of great interest for their potential applications in different fields [1–3]. This includes the recent use of a new class of fluorescent carbon nanomaterials called carbon nanodots (CNDs). These carbon-based nanomaterials have excellent properties: in particular, they have good water solubility and low toxicity, as well as high chemical stability. Hence, unlike semiconductor quantum dots [4, 5], these nanomaterials are biocompatible and not harmful to the environment. In recent years, CNDs have been actively investigated and widely used in interesting applications such as biosensing [6, 7]. In this context, they have been combined with electrochemiluminescence, leading to a new range of possibilities in the field of biosensing [8–10].

Electrogenerated chemiluminescence or electrochemiluminescence (ECL) is the generation of light occurring at the electrode when suitable molecules or ions are electrochemically activated and undergo highly exergonic electron-transfer reactions to form excited-state species (luminophores or emitters) that are capable of causing luminescence [11]. The mechanisms associated with these phenomena are well known and have been described in the literature, being classified into two main types [12, 13], the annihilation pathway and the co-reactant pathway. The latter is especially useful when charged radical ions are not stable enough for the ECL annihilation reaction or when one of the radical ions cannot be formed because the solvent has a narrow potential window.

The energy from the excitation source in ECL assays has a different nature than the signal, compared to fluorescence, giving ECL certain advantages such as a high signal to noise ratio. It therefore has great potential as a detection system for (bio)sensors [14, 15]. ECL-based biosensors have demonstrated remarkable properties and advantages such as controllability, high sensitivity, and low background noise. In fact, DNA sensing based on the detection of hybridization by ECL has drawn much attention [16–19]. Usually, the DNA acts as a co-reactant [20–24]. However, most of these sensors are not very sensitive.

Currently, substantial efforts are being made to improve the performance of these devices by exploring new materials and approaches with high sensitivity, high stability, and excellent controllability. In this sense, the use of CNDs opens new possibilities for the biosensor field, due to the optoelectronic properties of these nanomaterials. However, although some studies have described the use of CNDs as nanoemitters [25, 26] or co-reactants for the anodic ECL of tris(2,2′-bipyridyl)dichlororuthenium(II) ([Ru(bpy)_3_]^2+^) [27], the most widely used luminophore in this technique much remains to be explored in this regard.

MicroRNAs (miRNAs) are an important class of small non-coding RNA involved in the regulation of gene expression and whose mature products are around 18 − 25 nucleotides long. Their dysregulation has been associated with Alzheimer’s disease, cardiovascular diseases, and different types of cancer [28]. Thus, miRNAs can be used as biomarkers for early diagnosis and prognosis of these diseases. In particular, miRNAs are attractive biomarkers of response to cardiovascular diseases therapy given their stability, tissue-specific expression patterns, and secretion into bodily fluids [29]. For example, miRNA-21 levels were increased in fibroblasts in the failing heart [30, 31]. According to World Health Organization reports, cardiovascular diseases (CVDs) are almost the major causes of death globally and are responsible for over 18 million deaths every year. Therefore, diagnostic and prognostic biomarkers as miRNA-21 for CVDs have attracted significant interest in recent years [32]. Hence, over the past few decades, various miRNA sensing methods based on different techniques have been reported, such as optical [33], electrochemical [34], microfluidic [35], and ICP-MS [36]. However, detection and quantification of miRNA-21 using existing methods are relatively labor-intensive and time-consuming. Thus, the development of new rapid, simple, and effective methodologies for miRNA-21 detection is an area of great interest and the scientific community needs to put more emphasis on improving the early diagnosis methods. As consequence, nowadays, a great number of research works are focused on the development of new methodologies for rapid biomarker detection and recently several detection methods of miRNA employing new nanomaterials as CNDs, or graphene quantum dots have been reported [37–40]. In this sense, the combination of CNDs with ECL can be an interesting approach [41, 42] since these CNDs are employed to replace the traditional quantum dots as electrochemiluminescent luminophores. In addition to the simplicity of its synthesis and not requiring contaminating reagents, it presents advantages in terms of the analytical characteristics of the biosensor developed using this nanomaterial. In particular, for mRNA sensing, a low detection limit is achieved without the need of coupling more complicated methods, such as the isothermal circular strand-replacement polymerization (CSRP).

In this article, we describe a new sensitive method for the direct detection of miRNA-21. It consists of a new ECL biosensor based on water-soluble CNDs. We describe the synthesis of the CNDs, following environmentally friendly procedures, and their application as co-reactants to develop the ECL biosensor. Although some sensitive ECL biosensors for miRNA have previously been reported [43–46], they require complex and time-consuming development and labeling steps. Therefore, the search for new and quick methods for determining this biomarker is of great interest. Moreover, we do not use toxic reagents or time-consuming purification steps for the synthesis of these CNDs. Instead, we use natural products and non-toxic solvents.

**Scheme 1:**
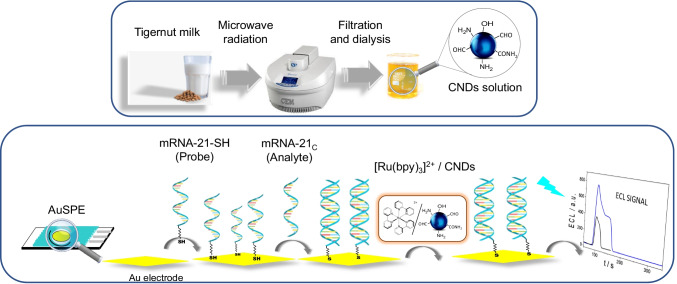
Steps of the friendly environmental chemistry procedure for CNDs synthesis and schematic representation of the DNA biosensor development: probe immobilization, hybridization with the analyte and ECL detection using [Ru(bpy)_3_]^2+^/CNDs system

The proposed biosensor does not require a complex procedure for its manufacture or complex labeling steps, as shown in Scheme 1. Briefly, the thiolated capture probe (miRNA-21-SH) is deposited on a disposable gold screen-printed electrode (AuSPE). Next, the analyte miRNA-21 is hybridized with the capture probe on the electrode surface. Finally, the hybridization is detected by the enhanced ECL emission of the [Ru(bpy)_3_]^2+^/CNDs system, allowing reliable sensing of miRNA-21. The method we have developed is highly sensitive, allowing fast, direct, and simple trace biomarker detection directly in serum samples from heart failure patients without previous RNA extraction neither amplification process.

## Experimental

### Chemicals

Tiger nut milk (from a local supermarket) was used as a natural precursor for the CNDs. Monobasic sodium phosphate (NaH_2_PO_4_·H_2_O), dibasic sodium phosphate (Na_2_HPO_4_·2H_2_O), sodium chloride (NaCl), hydrochloric acid (HCl), sodium hydroxide (NaOH), sulfuric acid (H_2_SO_4_), tris(2,2′-bipyridyl) dichlororuthenium (II) hexahydrate ([Ru(bpy)_3_]Cl_2_·6H_2_O), and human serum (from human male AB plasma, ref. H4522) were supplied by Merck (https://www.merckgroup.com/).

miRNeasy Serum/Plasma Advanced Kit (50), RNA Spike-in kit, UniRT, SYBR® Green master mix, UniSp2, LNA control primer set, UniRT, hsa-miR-103a-3p miRCURY LNA miRNA PCR Assay.cel-miR-39-3p miRCURY LNA miRNA PCR Assay and hsa-miR-21-5p LNA™ PCR primer set, UniRT were supplied by Qiagen (https://www.qiagen.com).

### Synthetic DNA oligonucleotides

Synthetic 22-mer oligonucleotides from Merck (https://www.merckgroup.com/) were used. A single-stranded DNA sequence modified at the 5′ end with a hexalkyl thiol was used as the capture probe, denoted miRNA-21-SH. For target analytes, a fully complementary sequence (denoted miRNA-21_C_) and a non-complementary sequence (denoted miRNA-21_NC_) were used. All sequences are listed in Table 1.

**Table 1:** Synthetic oligonucleotide sequences used

Nomenclature	Oligonucleotide sequence
miRNA-21-SH	5′-SH-(CH_2_)_6_–TCAACATCAGTCTGATAAGCTA
miRNA-21_C_	5′-UAGCUUAUCAGACUGAUGUUGA
miRNA-21_NC_	5′-AUCGAAUAGUCUGACUACAACU
miRNA-21_SM_	5′-UAGCUUAUC**T**GACUGAUGUUGA
miRNA-144	5´- UAACACUGUCUGGUAAAGAUGG
miRNA-155	5´- UUAAUGCUAAUCGUGAUAGGGGU

Stock solutions of the oligonucleotides corresponding to the complementary (miRNA-21_C_), non-complementary (miRNA-21_NC_), and single-mismatched (miRNA-21_SM_) sequences were prepared at a concentration of 100 µM in 10 mM phosphate buffer (PB) with 0.4 M NaCl, pH 7.0. Prior to use, thiol-modified probes were treated with DTT and then purified by elution through a NAP-10 column of Sephadex G-25. Afterwards, the stock solution of the thiolated miRNA-21-SH probe was prepared at a concentration of 9.00 µM in 10 mM PB, pH 7.0. Aliquots of a few microliters of all stock solutions were stored at − 20 °C.

### miRNA-21 serum samples from heart failure patients

miRNA-21 serum samples from heart failure patients were provided by IRICYS (Instituto Ramón y Cajal de Investigación Sanitaria) from the Biobank collection and correspond to the clinical study approved by the Local Ethics Committee (references 175/13 and 061/16) and complied with the Declaration of Helsinki. Serum clinical sample was obtained from whole blood samples by centrifugation, and aliquots of 250 µL were stored at − 80 °C. Two miRNA-21 serum clinical samples were used: serum 1 from heart failure patient with high levels of miRNA-21 expressed and serum 2 with low levels of miRNA-2, estimated by qRT-PCR at Ramon y Cajal Institute.

### Microscopic and spectroscopic techniques

The transmission electron microscope used was a JEOL JEM 2100HT operating at 200 kV. Images were processed using the Fiji software.

LECO CHNS-932 elemental analysis equipment and a FTIR (Fourier-transform infrared) Bruker IFS60v spectrophotometer were used.

UV–visible spectra were recorded from 200 to 800 nm in 1.0 cm quartz cells in a double-beam PharmaSpec UV-1700 series spectrophotometer from Shimadzu Corporation.

For fluorescence measurements, a Varian Cary Eclipse spectrofluorometer and quartz cuvettes with a 1-cm optical path were used.

X-ray photoelectron spectra (XPS spectra) of CNDs sample were obtained using a PHOIBOS 150 9MCD spectrometer (SPECS GmbH) equipped with a monochromatic Al Kα X-ray source operating at 200 W and 12 kV (1486.7 eV). Pass energies of 50 and 20 eV were used for acquiring both survey and high-resolution spectra, respectively. Survey data were acquired with an energy step of 1 eV and 100 ms dwell time per point. The high-resolu

tion scans were taken around the emission lines of interest with 0.1 eV steps and 100 ms dwell time per point.

Fluorescent microscopic images were taken on a Zeiss Axiovert 200 inverted microscope with a monochrome CCD camera and the Fiji software. Lumencor’s SPECTRA-X light engine was used. A 10 × /0.45 Plan-Apochromat Ph1 objective lens was used for detection. A DAPI filter (395/25) was employed, recording emissions with a DAPI filter (432/36).

Zeta potential measurements were determined at 25 °C using a Zetasizer Nano ZS instrument (Malvern Instrument Ltd., Grovewood, Worcestershire, UK).

### Electrochemical and electrochemiluminescence equipment

For electrochemical measurements, we used an Autolab/PGSTAT 10 potentiostat from Eco Chemie with the GPES 4.9 software. For electrochemiluminescence (ECL), a Metrohm DropSens ECL equipment was used.

Integrated screen-printed gold electrodes (4 mm diameter, AuSPEs) from DropSens S.L (Oviedo, Spain) were used as working electrodes. The format of these screen-printed electrodes includes a gold working electrode, a silver pseudo reference electrode, and a gold counter electrode. The electrodes were connected using a SPE Connector (DropSens S. L.) as interface. Sixty microliters of the sample was deposited onto the electrochemical cell to carrying out the electrochemical experiments (“drop test”). The electrochemical cell has not been degassed before carrying out the electrochemical experiments.

All material and solutions were sterilized in a Nüve OT012 autoclave before use.

### Procedures

#### Synthesis of the CNDs

In a typical synthesis, 3 mL of tiger nut milk was heated in a quartz flask under magnetic stirring for 30 min using a microwave reactor. The temperature and time were controlled and adjusted to maintain a constant power of 150 W and pressure of 170 psi [47]. These conditions produced yellow solutions. The solutions obtained were allowed to cool to room temperature. Next, they were filtered with a 0.45 µm nylon syringe filter and dialyzed with a Spectra/Por® 6 membrane (MWCO, 1 KDa) for one and a half hours to purify the synthesized samples. Finally, they were stored protected from light.

#### Preparation of microscope samples

The samples used for transmission electron microscopy (TEM) were prepared by depositing a 400 μM CNDs solution onto a carbon-film grid for direct observation after drying. To visualize the CNDs under the fluorescence microscope, 20 µL of a 40 µM solution of CNDs was placed on a slide and allowed to dry for 48 h.

#### Electrochemical pre-treatment of the electrode

AuSPEs were activated by a mild electrochemical pre-treatment that consisted of immersing them in a 0.5 M H_2_SO_4_ solution and applying ten successive cyclic potential sweeps at 0.1 V/s between − 0.30 and + 1.30 V.

#### Biosensor development

Biosensor development consists of three fundamental stages, as shown in Scheme 1, DNA capture probe immobilization, hybridization, and ECL detection.

##### Immobilization of the miRNA-21-SH probe by chemisorption of thiols

Ten microliters of the 9.00 µM solution of miRNA-21-SH in 10 mM PB was deposited onto the surfaces of clean, activated gold electrodes. After 72 h to complete evaporation, the modified electrodes were briefly immersed in purified water to remove non-adsorbed material [48].

##### Hybridization of the miRNA-21-SH probe with the miRNA-21

Ten microliters of a miRNA-21c solution (from 2.34 fM to 100.0 pM) was added onto the electrode surface modified with the thiolated miRNA-21-SH probe. Next, they were kept in a humid chamber at 40 °C for 1 h. Finally, they were briefly immersed in purified water to remove non-adsorbed material.

##### Electrochemiluminescent detection

Electrochemiluminescent measurements were performed by depositing 60.0 µL of 7 mM [Ru(bpy)_3_]^2+^ in 0.2 M PB, pH 8.0, with or without 70 µM CNDs, on the electrode surface modified with the hybridized miRNA samples.

To produce the ECL reaction, cyclic potential sweeps from 0.0 to 1.15 V for [Ru(bpy)_3_]^2+^ and from 0.0 to 1.30 V for [Ru(bpy)_3_]^2+^/CNDs were performed with a potential step of 0.002 V and a scan rate of 10 mV/s. Cyclic voltammograms and ECL spectra were recorded simultaneously.

#### miRNA-21 determination in spiked human serum samples

miRNA-21 was determined in human serum samples spiked with miRNA-21c to assess the applicability of the biosensor developed. Firstly, human serum spiked with miRNA-21c was prepared at a final concentration of 1.00 pM miRNA-21c (2.0 µL of 10.0 pM miRNA-21c solution was taken to a final volume of 20 µL with human serum). Then, 10.0 µL of this solution was added to the electrode surface modified with the thiolated miRNA-21-SH probe. Next, the hybridization process described above (in a humid chamber at 40 °C for 1 h) is developed. Finally, the electrodes were briefly immersed in purified water to remove non-adsorbed material. After this, ECL measurements were performed as described above, using a mixture of 7 mM [Ru(bpy)_3_]^2+^ and 70 µM CNDs in 0.2 M PB pH 8.0. Finally, the miRNA-21c concentration was estimated using the ECL average value obtained for the spiked human serum and the calibration plot (ECL = 29.1 log [miRNA-21_C_] + 722.3) (*R* = 0.9996), using the external calibration method, but subtracting the corresponding signal of the blank (the ECL signal of the diluted human serum without miRNA-21).

#### miRNA-21 determination in serum samples from heart failure patients

miRNA-21 was detected in serum samples from heart failure patients. Experimental procedures are described in detail in Electronic Supplementary Material (ESM).

## Results and discussion

### Choice of materials

Carbon nanodots (CNDs) have been chosen for the development of efficient DNA ECL biosensors, due to their biocompatibility and their property to act as co-reactants for the anodic ECL of [Ru(bpy)_3_]^2+^ [27].

### Synthesis and characterization of carbon nanodots

Photoluminescent CNDs were synthesized by carbonization of tiger nut milk using a microwave reactor synthesizer, as shown in Scheme 1 and explained in detail in the “Experimental” section. The temperature and reaction time were optimized using emissions from synthesized CNDs (see Fig. S1). Best results, considering the balance of speed and performance, were obtained with a reaction time of 30 min at a temperature of 200 °C, maintaining a constant pressure of 170 psi and a power of 150 W.

Hydrothermal treatments usually require carbonization for up to 18 h [49]. However, only 30 min is required with microwave radiation. Increasing the time of exposure to microwave radiation produces an increase in the emission intensity of the CNDs, which is more evident between 5 and 30 min (Fig. S1B). After 30 min, emissions remain almost constant, so this has been selected as the optimal synthesis time in terms of the fluorescence of the resulting product and the speed of the process.

The obtained CNDs were characterized by different techniques to elucidate their morphology, composition, and properties. Elemental analysis revealed that the CNDs contain 47.19% C, 5.75% H, 1.05% N (see Fig. S2), and 46.01% O (calculated). Zeta potential measurements gave an average value in aqueous solution of − 6.7 ± 0.3 mV. This negative value of zeta potential is probably due to the surface‐contained carboxylate groups on the CNDs.

#### Microscopic characterization

**Fig. 1:**
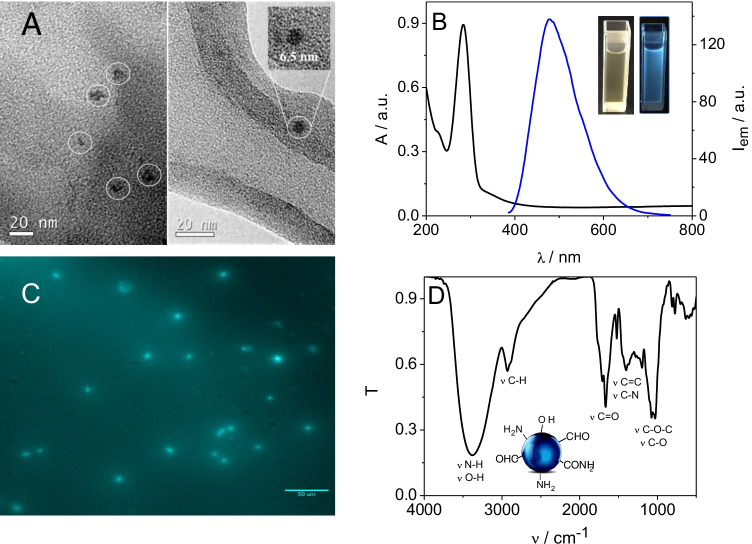
(**A**) TEM image of the synthesized CNDs. Inset: magnification of a CNDs TEM image. (**B**) Absorbance (black line) and fluorescence emission (blue line) spectra from 200 to 800 nm, the latter exciting at the maximum of excitation peak (ʎ = 380 nm). (**C**) Fluorescence micrograph and (**D**) FT-IR spectrum of the synthesized CNDs

The morphology and dimensions of the CNDs were determined by TEM. As shown in Fig. 1A, CNDs have a quasi-spherical morphology with diameters distributed in a range from 4 to 9 nm and an average size of 6.8 ± 1.5 nm (*n* = 35) (see histogram of Fig. S3).

#### Spectroscopic characterization

The UV–visible molecular absorption spectrum of the CNDs is shown in Fig. 1B. It can be seen that the CNDs have an absorption band at 284 nm that can be assigned to *n* → π* transitions from C = O groups and *π* → π* transitions from C = C groups [50]. This feature is characteristic of CNDs synthesized by carbonization of carbon-based materials [51] and demonstrates the presence of carbonyl groups on the surface of the synthesized CNDs. The fluorescence emission spectrum recorded by exciting CNDs at the maximum excitation peak (380 nm) presents a maximum at 478 nm (Fig. 1B). The emission peak decreases and shifts its position to a higher emission wavelength (from 471 to 550 nm) as *λ*_ex_ moves from 300 to 500 nm (∆*λ*_ex_ = 200 nm) (see Fig. S4). The redshift of the fluorescence emission can be attributed to differences in particle sizes and in the distribution of emissive sites on the surfaces of the CNDs. Smaller particles would be excited at shorter wavelengths than larger ones [52]. The fluorescence micrograph (Fig. 1C) again demonstrates the fluorescence of the CNDs, which appear as light spots.

The high carbon and oxygen content suggests that the particles obtained are nanometer-sized carbonaceous materials with a large number of carboxylic groups on the surface [49], a fact that can be confirmed by the FTIR spectrum (Fig. 1D). As can be seen, the spectrum exhibits the characteristic stretching band of OH and NH vibrations at 2900 and 3400 cm^−1^; a band located at 1650 cm^−1^ and attributed to C = O stretching vibration confirms that − COOH groups are present, explaining the high-water solubility of the CNDs. The band present at 2900 cm^−1^ can be assigned to C–H tension vibrations, and those above 1050 cm^−1^ are assigned to C–O and C–O–C bonds. The band at 1400 cm^−1^ probably corresponds to C–N or C = C vibrations. Therefore, carbonyl groups, alcohols, and amines are present in the synthesized CNDs.

X-ray photoelectron spectroscopy (XPS) studies were carried out to corroborate the results obtained from FTIR spectroscopy. The results revealed the presence of carbon, oxygen, nitrogen, phosphorus, and sodium, with the following percentage atomic concentrations: 64.4% C, 31.3% O, 3.9% N, 0.2% P, and 0.2% Na (Fig. S5A). Peak fitting of the C1s core level region (Fig. S5B) showed peaks at C = C (284.3 eV), C–C/C–H (284.8 eV), C–O/C–N (286.0 eV), C = O (287.6 eV), O–C = O (288.8 eV), and the characteristic π → π* vibrations of carbon atoms in graphene-like structures (292.4 eV). The O1s line fitting for the CNDs (Fig. S5C) shows two different chemical components centered at 531.5 eV (O = C) and 532.5 eV (O–C). The N1s spectrum (Fig. S5D) shows two peaks at 399.9 eV and 401.5 eV, which were attributed to the presence of C–N and N–H, respectively. The CND surface components determined by X-ray photoelectron spectroscopy are in good agreement with FTIR results.

The stability of the CNDs in aqueous solution was also studied following their fluorescence spectra. The results show that emission intensity remains constant for at least 30 days and then begins to decrease significantly (Fig. S6). We have also checked the reproducibility of the CND synthesis method. For this, the synthesis procedure has been repeated several times and no significant differences have been observed in the corresponding batches obtained, other than small differences in the concentration of CNDs. As we describe in detail in the “Electronic supplementary.material (ESM)” section, from the results obtained by elemental analysis, TEM, and UV–visible spectroscopy, we estimated the concentration of the synthesized CNDs. It was found to be of 400 µM, using the calculated molar extinction coefficient of 1.82 × 10^6^ M^−1^ cm^−1^ (see ESM and Fig. S7).

#### Electrochemical characterization

**Fig. 2:**
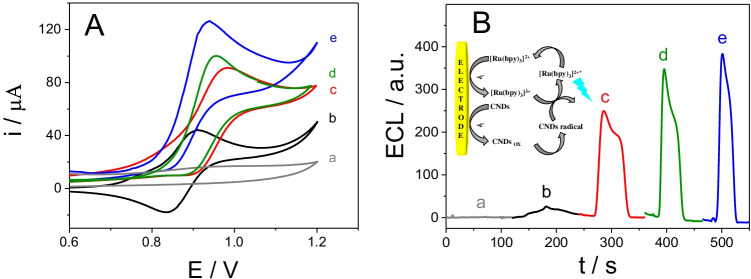
Cyclic voltammograms (**A**) and ECL signals (**B**) of a 70 µM CNDs (a) or 7 mM [Ru(bpy)_3_]^2+^ in the absence (b) and in the presence of 40 (c), 60 (d), and 70 µM CNDs (e) in 0.2 M PB, pH 8.0 at AuSPE from 0.6 to + 1.20 V (vs. Ag). Scan rate: 10 mVs^−1^. Inset: scheme of the [Ru(bpy)_3_]^2+^/CNDs ECL system

CNDs have been previously used as co-reactants in the anodic ECL of [Ru(bpy)_3_]^2+^ [27, 53]. To evaluate whether these synthesized CNDs could act as co-reactants in the anodic ECL of [Ru(bpy)_3_]^2+^, we recorded cyclic voltammograms of the CNDs, [Ru(bpy)_3_]^2+^, and [Ru(bpy)_3_]^2+^/CNDs in 0.2 M PB, pH 8.0, at an AuSPE. As can be seen in Fig. 2A, [Ru(bpy)_3_]^2+^ shows the characteristic redox process ascribed to the [Ru(bpy)_3_]^3+^/[Ru(bpy)_3_]^2+^ system, and the CNDs show no voltammperometric peaks within the potential window of the experiment. However, in presence of both CNDs and [Ru(bpy)_3_]^2+^, there is an electrocatalytic process, since the intensity of the [Ru(bpy)_3_]^2+^ oxidation peak increases concomitantly as the cathodic peak disappears; this was more evident on increasing the CND concentration from 40 to 70 µM (40, 60, and 70 µM) when a fixed concentration of 7.0 mM of [Ru(bpy)_3_]^2+^ was used.

Figure 2B shows that the ECL response of the [Ru(bpy)_3_]^2+^/CNDs mixture produced a strong ECL signal, which is consistent with that observed by cyclic voltammetry, whereas [Ru(bpy)_3_]^2+^ or CNDs alone exhibited weak ECL signals. Moreover, as observed by cyclic voltammetry, the ECL [Ru(bpy)_3_]^2+^ signal increased on increasing the concentration of CNDs.

We believe that CNDs act as co-reactants in the [Ru(bpy)_3_]^2+^/CNDs ECL system, being converted into reductive intermediates by the chemical oxidation of oxygen-containing units present on their surfaces by electrogenerated [Ru(bpy)_3_]^3+^ [27], similar to the role played by TPrA in the anodic ECL of the [Ru(bpy)_3_]^2+^/TPrA system [34]. Therefore, we propose the following typical “oxidative-reductive” co-reactant pathway mechanism for the [Ru(bpy)_3_]^2+^/CNDs ECL system:

$${\left[\mathrm{Ru}{\left(\mathrm{bpy}\right)}_{3}\right]}^{2+}-{\mathrm{e}}{-}\to {\left[\mathrm{Ru}{\left(\mathrm{bpy}\right)}_{3}\right]}^{3+}$$

$$\mathrm{CNDs}-{\mathrm{e}}{-}\to \left[\mathrm{CNDs}\right]^{.}+{\mathrm{H}}^{+}$$

$${\left[\mathrm{Ru}{\left(\mathrm{bpy}\right)}_{3}\right]}^{3+}+\left[\mathrm{CNDs}\right]^{.}\to {\left[\mathrm{Ru}{\left(\mathrm{bpy}\right)}_{3}\right]}^{2+*}$$

$${\left[\mathrm{Ru}{\left(\mathrm{bpy}\right)}_{3}\right]}^{2+*}\to {\left[\mathrm{Ru}{\left(\mathrm{bpy}\right)}_{3}\right]}^{2+}+\mathrm{hv}$$

[Ru(bpy)_3_]^2+^ and the CNDs are oxidized at the electrode surface to [Ru(bpy)_3_]^3+^ and the radical [CNDs], respectively. Then, [CNDs] reduces [Ru(bpy)_3_]^3+^ to [Ru(bpy)_3_]^2+^*, which is unstable and decays to the ground state, emitting a red light.

To investigate why CNDs assist in the anodic ECL of [Ru(bpy)_3_]^2+^, we obtained the UV–visible absorption and photoluminescent emission spectra of solutions of CNDs, [Ru(bpy)_3_]^2+^ and CNDs + [Ru(bpy)_3_]^2+^. The CNDs + [Ru(bpy)_3_]^2+^ mixture has the same absorption spectrum as [Ru(bpy)_3_]^2+^, with a negligible contribution from the CNDs (Fig. S8A). Something similar occurs in the case of the photoluminescent spectra (Fig. S8B), where [Ru(bpy)_3_]^2+^ and the CNDs maintain their original photoluminescence in the mixture. These results suggest that no reaction takes place between excited-state or ground-state CNDs and [Ru(bpy)_3_]^2+^ [35]. Hence, the light emission must be due to a reaction between the electrogenerated [Ru(bpy)_3_]^3+^ and the CNDs. [Ru(bpy)_3_]^2+^ is identified as the emitter in the mixture; therefore, the CNDs must be the co-reactant. Consequently, the ECL signal of [Ru(bpy)_3_]^2+^ increases on increasing the concentration of CNDs from 40 to 70 µM and then level off (Fig. 2B) from the inner filter effect of CNDs [27]. Based on these results, 70 µM of CNDs and 7.0 mM [Ru(bpy)_3_]^3+^ were chosen as optimal. Moreover, different pH values were also studied; the ECL signal of [Ru(bpy)_3_]^3+^ in 0.2 M PB increases with the pH up to 9.0. However, since biological samples will be used, pH 8.0 was chosen as more adequate. Thus, 70 µM of CNDs and 7.0 mM [Ru(bpy)_3_]^3+^ in 0.2 M PB, pH 8.0 were chosen as optimal conditions for further experiments.

### Electrochemiluminescent miRNA-21biosensor development

Based on the results described above, using the [Ru(bpy)_3_]^2+^/CNDs system, we developed an ECL DNA biosensor for the sensitive detection of miRNA-21 (Scheme 1). The [Ru(bpy)_3_]^2+^/CNDs ECL system was used to carry out the transduction that allows the biomarker miRNA-21 to be detected and quantified. Scheme 1 shows the different steps taken to design the biosensor.

As explained in the “Experimental” section, the first step is the immobilization of the miRNA-21-SH probe through the chemisorption of thiols on the surface of the AuSPE. In order to obtain a compact and standed-up thiolated probe monolayer, we have performed the immobilization either by direct adsorption of the capture probe alone for long time or employing mercaptohexanol (MCH) in addition to the thiolated probe. As was the case in previous works reported by us [54], best results were obtained when the thiolated probe was directly deposited on the electrode surface for long time (24 h). With this method, the hybridization effectiveness is higher since we observe the maximum difference between the ECL signal before and after hybridization of the probe with the target miRNA. These results agree well with those previously reported by us and confirm that immobilization time plays an important role in the self-assemble of the capture probe monolayer.

Our method allows also to determine the amount of thiolated oligonucleotide immobilized on the gold surfaces from the coulometry charge associated with the desorptive reduction of the miRNA-21-SH monolayer, as previously described for alkane thiols [55]. The surface coverage was calculated as 2.8 ± 0.2 × 10^−8^ mol oligonucleotide/cm^2^.

Next, hybridization of the probe with the complementary analyte sequence (10.0 pM miRNA-21_C_ solution) was performed on the electrode surface. Experimental parameters used, such as buffer, pH, and ionic strength for hybridization, are explained in detail in the “Experimental” section.

**Fig. 3:**
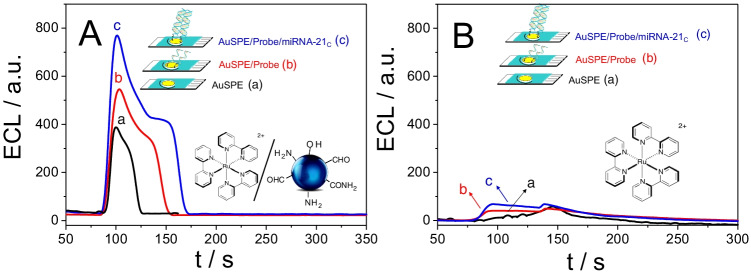
Cyclic voltammograms and ECL signal from 0.0 to + 1.3 V (vs. Ag) of a bare AuSPE (a) and an AuSPE modified with miRNA-21-SH (b) or miRNA-21C/miRNA-21-SH (c) using a 7 mM solution of [Ru(bpy)_3_]^2+^ in 0.2 M PB, pH 8.0, in the presence (**A**) and in the absence of 70 µM CNDs (**B**). Scan rate: 10 mVs^−1^

Finally, the hybridization event was detected via changes in the signal from the [Ru(bpy)_3_]^2+^/CNDs ECL system, after applying a cyclic potential sweep from 0.00 to + 1.30 V at 10 mVs^−1^. The resulting ECL signals were plotted as a function of time and compared to the bare electrode ECL signal (a). As can be seen in Fig. 3A, the biosensor response before hybridization (b) is less intense than that observed after hybridization with the fully complementary sequence (c). Specifically, hybridization of the probe produces an increase in the ECL signal of approximately 205 a.u. This can be explained by the formation of a stable helical conformation after hybridization, which enhances the electron-transfer reaction compared to the unstructured single-stranded unit. Therefore, these results demonstrate that the [Ru(bpy)_3_]^2+^/CNDs system used in the ECL technique can detect hybridization between oligonucleotides.

As a comparison, the same experiments were carried out using the [Ru(bpy)_3_]^2+^ complex alone (in the absence of CNDs). In this case, the ECL spectrum (Fig. 3B) shows that, after modifying the AuSPE with DNA, the signal increases slightly compared to that of the bare electrode. However, the difference in signals from the probe before and after hybridization with the complementary sequence is so small that it is difficult to discriminate between them.

From the above results, we concluded that the synthesized CNDs act as co-reactants for the ECL signal of [Ru(bpy)_3_]^2+^, improving the sensitivity of the method. Furthermore, in the presence of CNDs, there is a significant difference between the signals obtained before and after hybridization, which is essential for the development of a sensitive DNA biosensor.

**Fig. 4:**
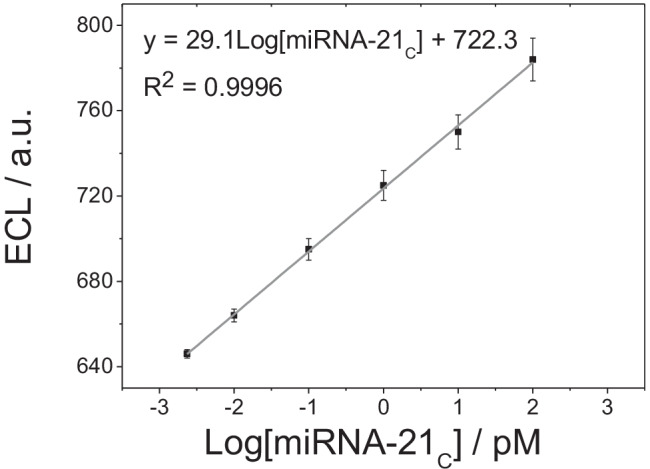
Calibration plot of the ECL biosensor response vs*.* miRNA-21_C_ concentration (from 10.0 fM to 100.0 pM)

After verifying the ability of the developed biosensor to detect the specific sequence of miRNA-21_C_, we studied its response to the biomarker at concentrations from 2.34 fM to 100.0 pM. The ECL signal increases linearly on increasing the target concentration, as can be seen in Fig. 4A. The plot of biosensor response versus log [miRNA-21_C_] fits the linear equation ECL = 29.1 log [miRNA-21_C_] + 722.3 (*R* = 0.9996). The method has a sensitivity of 29.1 a.u. log pM^−1^, obtained from the slope of the plot (Fig. 4B). The detection and quantification limits were found to be 0.721 (*S*/*N* = 3) and 2.34 (*S*/*N* = 10) fM, respectively.

Compared to other reported methods for the determination of miRNA-21, the ECL biosensor developed in this work exhibited a lower detection limit (Table 2). It also compares well with previously reported ECL biosensors and has much a wider linear concentration range. Furthermore, the method is simple, without the need for complex approaches or labeling steps.

**Table 2:** Analytical parameters of different methods for miRNA-21 detection

Detection technique	LOD (fM)	Linear range (fM to pM)	Ref
Colorimetric	3.2 × 10^6^	10 × 10^6^ to 0.98 10^6^	[56]
Fluorescent spectroscopy	4.2 × 10^3^	10 × 10^3^ to 2.0 × 10^3^	[57]
Electrochemical	30	100 to 2 × 10^3^	[58]
Electrochemical	1.5	5 to 2 × 10^3^	[59]
Organic electrochemical transistors	2 × 10^3^	5 × 10^3^ to 20 × 10^3^	[60]
ECL	0.65	1.0 to 100	[44]
ECL	0.03	0.1 to 10	[41]
ECL	2.78	0.10 to 100	[39]
ECL	0.721	2.34 to 100	This work

The selectivity of the ECL biosensor was tested using a non-complementary sequence (miRNA-21_NC_) and a single mismatched sequence (miRNA-21_SM_). In addition, in order to consider the high homology between miRNA families, the biosensor response to a miRNA144 and miRNA-155 sequence was tested (see Fig. S9). For this purpose, ECL biosensor responses were recorded before and after hybridization with a 100.0 pM solution of a non-complementary (miRNA-21_NC_), a single-mismatched (miRNA-21_SM_), interferent sequences (miRNA144 and miRNA-155), or a totally complementary sequence (miRNA-21_C_) used as control. When the probe is hybridized with the complementary sequence, an increase in ECL signal of around 205 ± 8 a.u. is observed. However, when hybridization takes place with the mismatched sequence, the increase in the ECL signal is lower (around 125 ± 7 a.u.). The hybridization with the single-mismatched target will give a distorted double-helix, causing a decrease in the ECL signal. In the case of the non-complementary and interferent sequences (miRNA-144 and miRNA-155), a response very similar to that of the probe is observed. This result suggests that no hybridization process is taking place; the small increase observed is probably due to a nonspecific adsorption. The reproducibility of the method was estimated from five devices prepared using the same protocol. In all cases, the relative standard deviation (RSD) of the ECL signal was found to be less than 7%. Moreover, the biosensor can detect the target miRNA-21_C_ over a period of 1 month.

### miRNA-21 determination in spiked human serum samples

Finally, we evaluated the applicability of the biosensor by determining miRNA-21 in human serum samples using an external calibration method, as described in detail the “Experimental” section. ECL measurements gave an average value of 723.8 a.u. From the calibration plot, the miRNA-21 concentration in the spiked serum sample (final miRNA-21 concentration of 1.00 pM) was found to be 1.13 pM with a recovery of 113% and a RSD of 4%. This result demonstrates that the proposed biosensor can be used for practical applications and has great potential as an alternative to the classical methods for detecting miRNA-21 in human serum samples.

### miRNA-21 determination in serum samples from heart failure patients

Based on the great interest of having simple methodologies for rapid biomarker detection and considering the good results obtained with the developed biosensor in spiked human serum samples, we take a step forward and applied our methodology to detect miRNA-21 directly in serum samples from heart failure patients, provided and qRT-PCR checked by IRICYS (Instituto Ramón y Cajal de Investigación Sanitaria), as we described in detail in the “Experimental” section. Two different types of serums were analyzed (serum 1 and serum 2, latest used as control). It is worth to note that in the case of qRT-PCR method, it is necessary to include a RNA extraction step before qRT-PCR analysis and the whole process requires long time of analysis. The developed ECL biosensors only require a denaturation step (see the “Experimental” section) before the analysis. The ECL biosensor response to the serum 1 (Fig. S10) shows a signal of 724 ± 10 a.u. compared to the signal of the probe (575 ± 6 a.u.). The concentration was estimated using the calibration plot (ECL = 29.1 log [miRNA-21_C_] + 722.3). A concentration of 1.14 pM was obtained. As we describe in the “Experimental” section, serum 2 was used as control. In this case, the biosensor response to serum 2 shows a signal of 600 ± 8 a.u., suggesting that the miRNA-21 concentration is extremely low and no miRNA-21 overexpression is observed. These results agree well with those obtained by qRT-PCR which renders Ct = 23 in serum sample 1 and Ct = 31 in serum sample 2. Normalizer and controls (miR103, UniSp2, and cel 39) did not show differences in Ct between both samples.

Based on the above results, we can conclude that the biosensor developed can detect a miRNA-21 sequence directly in clinical human serum samples from heart failure patients without any previous amplification process. It is a simple, sensitive, fast, and scalable tool for detecting this biomarker, allowing early detection of the disease and increasing the probability of patient survival.

## Conclusions

CNDs have been synthesized through an environment friendly chemistry-based procedure using tiger nut milk as a natural precursor and have been demonstrated to be excellent co-reactants for the development of an improved [Ru(bpy)_3_]^2+^ ECL DNA biosensor. The [Ru(bpy)_3_]^2+^/CNDs system was used for the first time to detect the specific sequence of a biomarker associated with breast cancer, miRNA-21. Electrochemical studies have confirmed that CNDs catalyze the oxidation of the [Ru(bpy)_3_]^2+^ complex on DNA-modified AuSPEs, improving the ECL signal. The [Ru(bpy)_3_]^2+^/CNDs system is highly promising for bioanalysis, as exemplified in this work by the detection of a cancer biomarker in spiked human serum samples as well in clinical serum samples from heart failure patients without any previous amplification process. This work has opened new approaches for the synthesis of CNDs and has deepened and broadened our knowledge, as well as the application of these nanomaterials for biosensing, which will be beneficial for further development based on CNDs.

## Supplementary Information

Below is the link to the electronic supplementary material.

Supplementary file1 (DOCX 4710 KB)
