# Supplementary material for: Carbon nanodot–based electrogenerated chemiluminescence biosensor for miRNA-21 detection
Source: Mikrochim Acta. 2021 Oct 30;188(11):398. doi: 10.1007/s00604-021-05038-y (PMC8557186; doi:10.1007/s00604-021-05038-y)
Supplement: Supplementary file 1 — Supplementary file1 (DOCX 4710 KB) [file 604_2021_5038_MOESM1_ESM.docx]

**Electronic Supplementary Material**

**Carbon nanodots–based electrogenerated chemiluminescence biosensor for miRNA-21detection**

Laura Gutiérrez-Gálvez^1^, Tania García-Mendiola^1,2,3,^*, Cristina Gutiérrez-Sánchez^1,*^ Tamara Guerrero-Esteban^1^, Cristina García-Diego^4^, Irene Buendía^5^, M. Laura García-Bermejo^5^, Félix Pariente^1,2^ and Encarnación Lorenzo^1,2,3^.

^1^ Department of Analytical Chemistry and Instrumental Analysis and ^2^ Institute for Advanced Research in Chemical Sciences (IAdChem), Autonomous University of Madrid, Ciudad Universitaria de Cantoblanco, 28049 Madrid, Spain.

^3^ IMDEA Nanociencia, Ciudad Universitaria de Cantoblanco, 28049, Madrid, Spain.

^4^ Instituto de Catálisis y Petroleoquímica, Consejo Superior de Investigaciones Científicas, C/Marie Curie 2, 28049 Madrid, Spain.

^5^ Biomarkers and Therapeutic Targets Group and Core Facility, Instituto Ramón y Cajal de Investigación Sanitaria (IRYCIS), Spanish Renal Research Network (REDinREN), Madrid, Spain.

*Corresponding author. Tel: +34 91 4976024/8426. E-mail: [cristina.gutierrezs@uam.es](mailto:cristina.gutierrezs@uam.es), [tania.garcia@uam.es](mailto:tania.garcia@uam.es)

**Experimental**

**Equipment**

The microwave synthesis reactor used to synthesize the CNDs was a CEM Discover LabMate™ supplied by CEM. 10 mL syringes, 0.45 µm nylon syringe filters and Spectra/Por® 6 dialysis membranes (MWCO, 1 KDa) provided by General Laboratory Supplies (SGL) were used to purify the synthesized CNDs. A Thermal Cycler (Applied Biosystem) and Light Cycler 480 (Roche) were used for clinical PCR samples preparation.

**Procedures**

**miRNA-21 determination in serum samples from heart failure patients.**

miRNA-21 was detected in serum samples from heart failure patients by means of two different strategies: the developed ECL biosensor and qRT-PCR used to validate the biosensor. For this purpose, aliquots of the same sample were used for both methodologies.

ECL biosensor miRNA-21 determination

Clinical samples were just denatured (by heating until 100 °C followed by rapid cooling) before their analysis. Then 5.0 µL of the denatured samples were deposited on the biosensor surface to proceed with the hybridization and ECL measurements described above for spiked human serum.

qRT-PCR miRNA-21 determination

Before miRNA-21 analysis in clinical samples by qRT-PCR, RNA was extracted using an aliquot of 250 µL of the serum samples from heart failure patients. Prior to RNA isolation, a synthetic RNA (spike-in) was added to clinical serum samples and served as a technical control of extraction homogeneity by further spike-in amplification. Isolation of total RNA enriched in miRNAs was performed using the miniRNA easy kit (Qiagen) and 200 µL of clinical serum. An external RNA (cell-miR-39) was added and further amplified as a control of cDNA synthesis efficiency. The Universal RT miRNA PCR System (Qiagen) was used for cDNA synthesis. Briefly, 3 µL of RNA was used as a template for PCR reaction in a final volume of 20 µL of clinical serum samples. cDNA was diluted 1/11 with nuclease-free sterile water, and 4 µL was used as a template for PCR reactions. qRT-PCR detection was performed using SYBR Green and specific LNA probes for each selected miRNA (Qiagen). All reactions were carried out in triplicate using Light Cycler 480 (Roche), and Ct values were calculated using the second derivative method (Light Cycler 480 Software 1.5, Roche, Basel, Switzerland). miRNA expression values are presented as ∆Ct, obtained as follows: ∆Ct = miRNA Ct − housekeeping Ct. miR103a-3p was used as normalizer, being Ct (crossing threshold) the cycle from which amplified template is detected.

**Fig. S1** A) Fluorescence emission spectra of the CNDs synthesized applying a radiation microwave time of 30 minutes at temperatures of 100 and 200 ºC. B) Bar diagram of the CNDs emission at different radiation microwave time (5, 10, 15, 30, 60, 90 and 120 min), at a temperature of 200 ºC, pressure of 170 psi and 150 W of power.


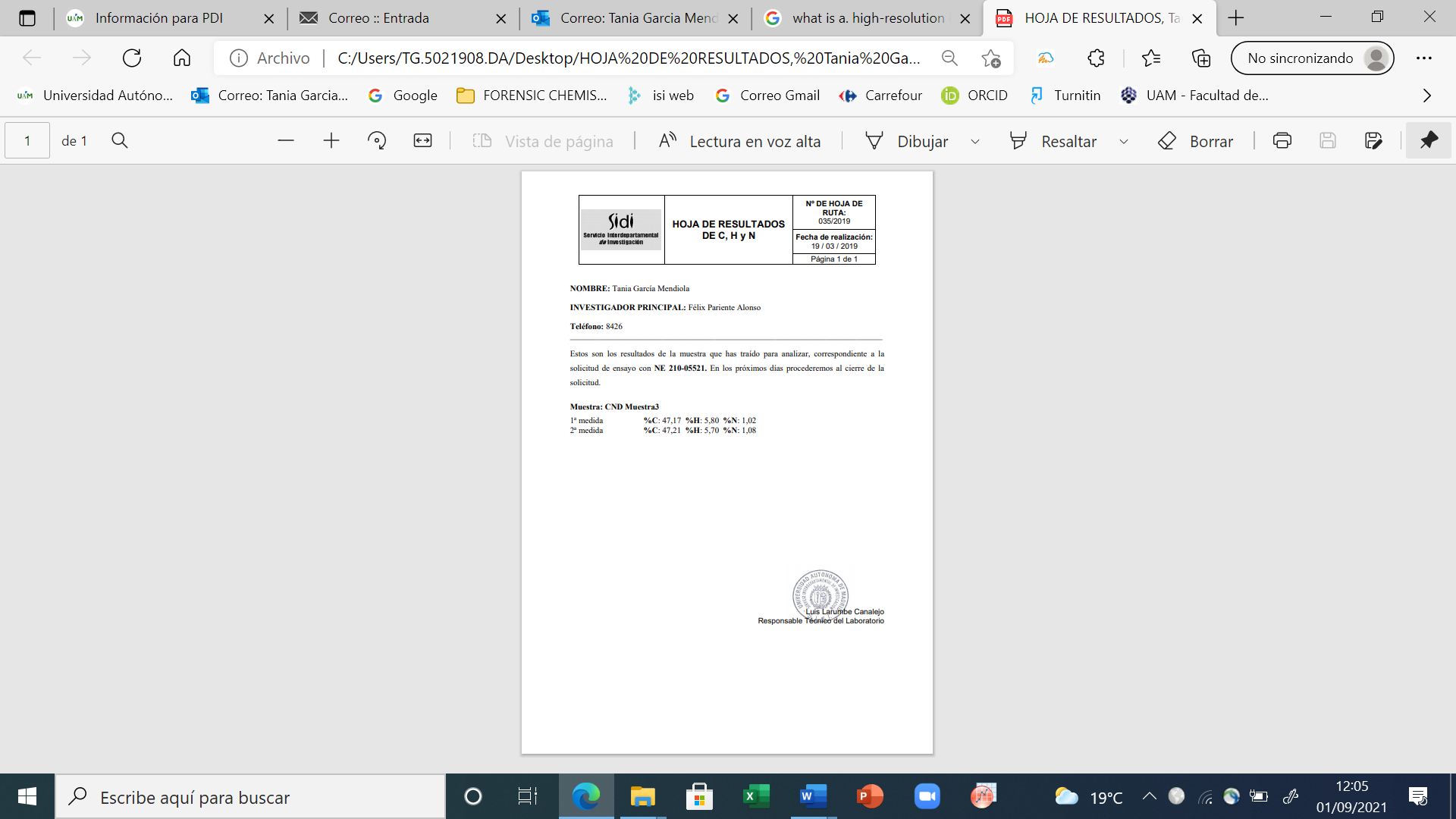


**Fig. S2** Image of the report obtained in the elemental analysis of CNDs.

**Fig. S3** Histogram profile of CNDs size distribution.

**Fig. S4** Fluorescence emission spectra of the CNDs at different excitation wavelengths: from 300 nm to 500 nm.

**Fig. S5** XPS survey spectra of the synthesized CNDs (A). Deconvolution of B) C1s; C) O1s and D) N1s core-levels region of the CNDs.

**Fig. S6**. Plot of the fluorescence emission of the CNDs *vs*. time (days).

***1.Estimation of the CNDs concentration***

The estimation of CNDs concentration and its molar extinction coefficient was carried out following a similar procedure described by Femig et al. for gold nanoparticles. [^1^](#_ENREF_1) Firstly, we obtain the number of carbon atoms in the sample through the mass of the initial reagents and the percentage by mass of carbon achieved in the elemental analysis. From this result, the number of CNDs that would be in the solution can be calculated, taking into account the volume relationship between a carbon atom and the CNDs and assuming they are spherical. Finally, dividing by the volume of the solution obtained after synthesis, a concentration of 400 µM have been estimated. Moreover, the molar extinction coefficient (ε) of them at their maximum absorption (λ_max_ = 284 nm) was calculated. The absorption spectra of the CNDs at different concentrations are shown in **Fig. S5**, from which the linear fit represented in the inset of **Fig. S5** can be extracted, obtaining an estimated value of ε_284nm_ = 1.82 · 10^6^ M^-1^ cm^-1^.

**Fig. S7**. Absorption spectra at different concentrations of CNDs (0.04 μM, 0.10 μM, 0.14 μM, 0.20 μM, 0.30 μM, 0.34 μM, 0.40 μM, 0.50 μM and 0.55 μM). Inset. Plot of the absorbance *vs*. CNDs concentration.

**Fig. S8**. UV-visible absorption (A) and emission (B) spectra of [Ru(bpy)_3_]^2+^(a) CNDs (b) and [Ru(bpy)_3_]^2+^/CNDs (c), respectively.

**Fig. S9** ECL bar diagrams of AuSPE modified with the probe (miRNA-21-SH) before (a) and after the hybridization with a 100.0 pM solution of: miRNA-144 (b) and miRNA-155 (c) sequences, non-complementary sequence, mRNA-21_NC_ (d), a single-mismatched sequence, miRNA-21_SM_ (e), and a complementary sequence, miRNA-21_C_ (f).

**Fig. S10**. ECL bar diagrams of AuSPE modified with the probe (miRNA-21-SH) before (a) and after the hybridization with the serum sample 2 (control) (b) and serum sample 1(c) from heart failure patients.

**References**

1. W. Haiss, N. T. K. Thanh, J. Aveyard and D. G. Fernig, *Analytical Chemistry*, 2007, **79**, 4215-4221.
